# Large language model answers medical questions about standard pathology reports

**DOI:** 10.3389/fmed.2024.1402457

**Published:** 2024-09-18

**Authors:** Anqi Wang, Jieli Zhou, Peng Zhang, Haotian Cao, Hongyi Xin, Xinyun Xu, Haiyang Zhou

**Affiliations:** ^1^Division of Colorectal Surgery, Changzheng Hospital, Navy Medical University, Shanghai, China; ^2^UM-SJTU Joint Institute, Shanghai Jiao Tong University, Shanghai, China; ^3^Department of Pathology, Changzheng Hospital, Navy Medical University, Shanghai, China; ^4^Division of Breast and Thyroid Surgery, Changzheng Hospital, Navy Medical University, Shanghai, China

**Keywords:** large language model, medical question, pathology report, colorectal cancer, Generative Pretrained Transformer

## Abstract

This study aims to evaluate the feasibility of large language model (LLM) in answering pathology questions based on pathology reports (PRs) of colorectal cancer (CRC). Four common questions (CQs) and corresponding answers about pathology were retrieved from public webpages. These questions were input as prompts for Chat Generative Pretrained Transformer (ChatGPT) (gpt-3.5-turbo). The quality indicators (understanding, scientificity, satisfaction) of all answers were evaluated by gastroenterologists. Standard PRs from 5 CRC patients who received radical surgeries in Shanghai Changzheng Hospital were selected. Six report questions (RQs) and corresponding answers were generated by a gastroenterologist and a pathologist. We developed an interactive PRs interpretation system which allows users to upload standard PRs as JPG images. Then the ChatGPT's responses to the RQs were generated. The quality indicators of all answers were evaluated by gastroenterologists and out-patients. As for CQs, gastroenterologists rated AI answers similarly to non-AI answers in understanding, scientificity, and satisfaction. As for RQ1-3, gastroenterologists and patients rated the AI mean scores higher than non-AI scores among the quality indicators. However, as for RQ4-6, gastroenterologists rated the AI mean scores lower than non-AI scores in understanding and satisfaction. In RQ4, gastroenterologists rated the AI scores lower than non-AI scores in scientificity (*P* = 0.011); patients rated the AI scores lower than non-AI scores in understanding (*P* = 0.004) and satisfaction (*P* = 0.011). In conclusion, LLM could generate credible answers to common pathology questions and conceptual questions on the PRs. It holds great potential in improving doctor-patient communication.

## Introduction

Large language model (LLM) combines the power of deep learning with transformer architectures to understand and generate human language, showing potential in answering medical questions (1, 2). The current LLM has been found to be prone to errors in the specialty, leading to limited efficacy in clinical practice (3). Nonetheless, several studies showed LLM can effectively convey health information and generate answers of higher quality and greater empathy compared to those produced by doctors (4, 5). Pathology reports (PRs) which contain information for diagnostic evaluation and clinical decision making are often hard to understand for patients (6–8). Incorporating these cost-effective technological solutions into clinical practice has the potential to mitigate disparities and provide particular benefits to patients from socioeconomically disadvantaged backgrounds, who typically exhibit lower levels of health literacy (9). In this study, we evaluated the ability of LLM to answer pathology questions based on PRs of colorectal cancer (CRC). These efforts take a patient-centric approach and aim to holistically evaluate LLM-based systems' ability to explain complex medical reports to patients, which distinguishes our study from previous works.

## Materials and methods

### LLM answers common pathology questions

Four common questions (CQs) and corresponding answers (1 question matches 2 answers) about pathology were retrieved based on their frequency and relevance from publicly available medical resources including reputable medical websites, online health forums, and pathology textbooks. These questions were input as prompts for Chat Generative Pretrained Transformer (ChatGPT) (gpt-3.5-turbo) twice on the same day, and the answers were recorded as artificial intelligence (AI1 and AI2), respectively. All the answers were anonymized to prevent any identification of the generation time or sequence, ensuring an unbiased evaluation. The quality indicators (understanding, scientificity, satisfaction) of 16 answers were evaluated by six gastroenterologists (three senior gastroenterologists and three fellows) on a 7-point Likert scale (10). The answers were randomized before being presented to the gastroenterologists to avoid potential order effects. The text similarity among all answers were compared using Jaccard Similarity (11). The raters were also asked to determine whether the answers were generated by AI or not.

### LLM answers pathology questions based on PRs

Standard PRs from 5 CRC patients who received radical surgeries in Shanghai Changzheng Hospital between January 1, 2022, and December 31, 2022 were selected. These patients were selected based on the criteria of being free from distant metastasis. The selection of these reports aimed to ensure a diverse yet representative sample of standard PRs in CRC. Six report questions (RQs) were developed for each PR, focus on pathological type, pathological stage, immunohistochemical result, adjuvant therapy, prognosis, and follow-up. The corresponding answers (1 question matches 1 answer) were generated by a gastroenterologist and a pathologist based on National Comprehensive Cancer Network Guidelines (Version 3. 2023). We developed an interactive PRs interpretation system which allows users to upload standard PRs of CRC as JPG images (http://pathology.doctorhealthx.com). We used optical character recognition to digitalize the uploaded reports and convert image data into text format and then prompt ChatGPT questions based on the reports (12, 13). ChatGPT's responses to these questions were generated, recorded, and carefully reviewed to ensure accurate representation of the original reports. The quality of 60 answers were evaluated by six gastroenterologists and seven out-patients using the 7-point Likert scale.

### Statistical analysis

Data was shown as mean (standard deviation). We used Mann-Whitney *U* test to compare the quality indicator and readability of answers. We used the readability metric from a text analysis package “cntext” to calculate the text complexity of Chinese, measured by the average number of characters in each clause and the proportion of verbs and conjunctions in each sentence (14). Sensitivity, specificity, positive predictive value, negative predictive value, and accuracy were calculated to evaluate raters' performance in detecting AI-generated answers. All analyses were accomplished using software SPSS (version 25), and 2-sided *P* < 0.05 was considered statistically significant.

## Results

### Performance of answering CQs

As for CQs, the AI answers had low text similarity (10%-23%) compared to non-AI answers, and the text similarity ranged from 30% to 42% between the 2 AI answers (Table 1). The gastroenterologists demonstrated 56.9% accuracy in identifying AI answers, with 45.8% sensitivity and 62.5% specificity. Fellows showed 50.0% accuracy and 33.3% sensitivity (Table 2). Gastroenterologists rated AI answers similarly to non-AI answers in understanding (AI, 4.5–5.7 vs. non-AI, 4.7–5.3, nonsignificant), with the AI mean scores higher than non-AI scores in the CQ2 and 4. Scientific adequacy scores were also similar (AI, 4.8–5.7 vs. non-AI, 4.7–5.3, nonsignificant), with the AI mean scores higher than non-AI scores, except for the CQ3. AI and non-AI received similar scores regarding satisfaction with the answers (AI, 5.2–5.7 vs. non-AI, 4.4–5.1, nonsignificant), with the AI mean scores higher than non-AI scores (Table 3).

**Table 1:** Comparison of answers to four common questions about pathological examination.

**CQs**		**AI1 vs. AI2**		**AI1 vs. Hospital1**		**AI1 vs. Hospital2**		**AI2 vs. Hospital1**		**AI2 vs. Hospital2**	
		**Words**	**Match, %**	**Words**	**Match, %**	**Words**	**Match, %**	**Words**	**Match, %**	**Words**	**Match, %**
CQ1	What is a pathological examination?	298 vs. 342	42	298 vs. 299	15	298 vs. 321	14	342 vs. 299	16	342 vs. 321	15
CQ2	Why is a pathological examination performed?	494 vs. 596	30	494 vs. 504	23	494 vs. 252	20	596 vs. 504	22	596 vs. 252	15
CQ3	What to constitute a pathological report?	618 vs. 409	31	618 vs. 452	23	618 vs. 527	20	409 vs. 452	23	409 vs. 527	21
CQ4	What is an immunohistochemical result?	463 vs. 354	31	463 vs. 97	12	463 vs. 268	16	354 vs. 97	10	354 vs. 268	12

**Table 2:** Performance of four interpreters in detecting answers of CQs generated by AI.

**Detecting answers of CQs generated by AI**	**Sensitivity, %**	**Specificity, %**	**PPV, %**	**NPV, %**	**Accuracy, %**
All interpreters (*n* = 6)	45.8	62.5	37.9	69.8	56.9
Senior gastroenterologists (*n* = 3)	58.3	66.7	46.7	76.2	63.9
Fellows (*n* = 3)	33.3	58.3	28.6	63.6	50.0

**Table 3:** Quality indicators for answers to common question (CQ) and report question (RQ) from AI and from non-AI sources.

**Pathology question**		**Source of answers**	***“The answers are easy to understand.”***				***“The answers are scientifically adequate.”***		***“I am satisfied with the answers.”***			
			**Mean (SD) gastroenterologists**	***P***	**Mean (SD) patients**	***P***	**Mean (SD) gastroenterologists**	***P***	**Mean (SD) gastroenterologists**	***P***	**Mean (SD) patients**	***P***
CQ1	What is a pathological examination?	AI	4.5 (1.2)	0.469	NA	NA	5.2 (1.5)	0.704	5.2 (1.8)	0.439	NA	NA
		Non-AI	5.0 (1.5)				4.9 (1.2)		4.7 (1.4)			
CQ2	Why is a pathological examination performed?	AI	5.3 (1.2)	0.683			5.7 (1.0)	0.208	5.7 (1.0)	0.167		
		Non-AI	4.9 (1.6)				4.8 (1.3)		4.8 (1.1)			
CQ3	What to constitute a pathological report?	AI	4.8 (1.5)	0.561			4.8 (2.0)	0.712	5.2 (1.8)	0.672		
		Non-AI	5.3 (1.3)				5.3 (1.5)		5.1 (1.3)			
CQ4	What is an immunohistochemical result?	AI	5.7 (1.5)	0.145			5.5 (0.8)	0.171	5.7 (1.5)	0.054		
		Non-AI	4.7 (1.2)				4.7 (1.3)		4.4 (1.0)			
RQ1	Summary of pathological type of tumor.	AI	5.8 (0.6)	0.212	5.3 (1.1)	0.002	6.2 (0.5)	0.011	6.6 (0.5)	0.000	5.3 (0.9)	0.000
		Non-AI	5.5 (0.6)		4.5 (1.2)		5.8 (0.6)		5.8 (0.5)		4.2 (1.2)	
RQ2	Interpretation of pathological stage.	AI	6.0 (0.6)	0.000	5.1 (1.2)	0.000	6.0 (0.7)	0.031	6.3 (0.4)	0.000	5.4 (1.1)	0.000
		Non-AI	5.0 (0.7)		3.8 (1.2)		5.4 (0.9)		5.4 (0.8)		3.7 (1.6)	
RQ3	Interpretation of immunohistochemical results.	AI	5.8 (0.5)	0.155	4.5 (1.3)	0.009	6.0 (0.0)	0.106	6.2 (0.6)	0.014	5.0 (1.2)	0.015
		Non-AI	5.6 (0.5)		3.7 (1.2)		5.8 (0.4)		5.6 (0.8)		4.3 (0.9)	
RQ4	How to choose adjuvant therapy?	AI	5.6 (0.8)	0.012	4.6 (1.0)	0.004	5.4 (1.0)	0.011	5.3 (0.8)	0.000	4.5 (1.4)	0.011
		Non-AI	6.3 (0.6)		5.4 (1.3)		6.2 (0.7)		6.3 (0.6)		5.3 (1.4)	
RQ5	Prediction of prognosis.	AI	5.6 (0.6)	0.076	5.2 (0.9)	0.220	6.0 (0.8)	0.998	5.5 (0.6)	0.037	5.1 (1.1)	0.901
		Non-AI	6.0 (0.6)		5.3 (1.4)		5.9 (0.7)		5.9 (0.6)		4.7 (1.8)	
RQ6	Advice for follow-up.	AI	5.7 (0.8)	0.017	5.1 (0.9)	0.298	6.2 (0.6)	0.999	5.7 (0.9)	0.005	5.2 (1.2)	0.792
		Non-AI	6.3 (0.6)		5.1 (1.4)		6.2 (0.6)		6.3 (0.6)		4.9 (1.5)	

Interpreted by 6 gastroenterologists and 7 patients with 7-points Likert Scale (7 = Strongly agree, 1 = strongly disagree).

Interpretation 1: “The answers are easy to understand.”

Interpretation 2: “The answers are scientifically adequate.”

Interpretation 3: “I am satisfied with the answers.”

Statistical analysis by Mann Whitney U test. P < 0.05 as significant.

### Performance of answering questions based on PRs

As for RQ1-3, gastroenterologists rated the AI mean scores higher than non-AI scores in understanding (AI, 5.8–6.0 vs. non-AI, 5.0–5.6, significant in RQ2), scientific adequacy (AI, 6.0–6.2 vs. non-AI, 5.4–5.8, significant in RQ1 and 2), and satisfaction (AI, 6.2–6.6 vs. non-AI, 5.4–5.8, significant in RQ1-3). Similarly, patients rated the AI mean scores higher than non-AI scores in understanding (AI, 4.5–5.3 vs. non-AI, 3.7–4.5, significant in RQ1-3) and satisfaction (AI, 5.0–5.4 vs. non-AI, 3.7–4.3, significant in RQ1-3). However, as for RQ4-6, gastroenterologists rated the AI mean scores lower than non-AI scores in understanding (AI, 5.6–5.7 vs. non-AI, 6.0–6.3, significant in RQ4 and 6) and satisfaction (AI, 5.3–5.7 vs. non-AI, 5.9–6.3, significant in RQ4-6). In RQ4, gastroenterologists rated the AI mean scores lower than non-AI scores in scientific adequacy (AI, 5.4 vs. non-AI, 6.2, *P* = 0.011); patients rated the AI mean scores lower than non-AI scores in understanding (AI, 4.6 vs. non-AI, 5.4, *P* = 0.004) and satisfaction (AI, 4.5 vs. non-AI, 5.3, *P* = 0.011) (Table 3).

### Performance of readability

Overall, AI answers had more words than non-AI answers (AI, 338.1 vs. non-AI, 179.7). The complexity levels were a little higher for AI answers, with readability scores lower than non-AI answers (AI, 18.4 vs. non-AI, 21.5), although no statistical significance was found. In answering CQs, AI answers had lower readability scores than non-AI answers (AI, 18.1 vs. non-AI, 29.7, *P* = 0.005). In answering RQs, AI had lower readability scores in RQ2, 3, 4, and 6, but no significant statistical differences were found except in RQ3 (*P* = 0.008); AI had higher readability scores in RQ1 and 5, with significant statistical differences (*P* = 0.021 and 0.008). This could be attributed to the overall long lengths of the AI answers (Table 4).

**Table 4:** Comparison of readability scores of answers from AI and non-AI.

**Sources of answers**		**Words, mean (SD)**	**Readability score, mean (SD)**	***P***
All pathology questions	AI	338.1 (156.9)	18.4 (3.5)	0.069
	Non-AI	179.7 (180.2)	21.5 (8.7)	
CQs	AI	447.6 (110.9)	18.1 (2.3)	0.005
	Non-AI	340.6 (136.6)	29.7 (9.7)	
RQ1	AI	69.4 (36.8)	16.4 (3.0)	0.021
	Non-AI	17.8 (2.7)	8.9 (1.4)	
RQ2	AI	202.4 (23.5)	15.6 (2.6)	0.094
	Non-AI	37.0 (1.8)	18.5 (0.9)	
RQ3	AI	524 (79.6)	17.4 (4.9)	0.008
	Non-AI	469.8 (119.2)	29.1 (1.1)	
RQ4	AI	322.8 (40.7)	19.3 (1.3)	0.140
	Non-AI	154.0 (67.4)	21.6 (5.8)	
RQ5	AI	319.2 (76.5)	21.6 (3.0)	0.008
	Non-AI	47.2 (24.0)	14.4 (1.5)	
RQ6	AI	415.4 (26.4)	20.5 (2.0)	0.110
	Non-AI	95.0 (0.0)	23.3 (0.0)	

Readability is measured by the complexity of the text; a higher score signifies a more complex text and thus, lower readability. Statistical analyses between the AI and non-AI answers were done with the Mann-Whitney U test tests.

## Discussion

The text similarity results suggested the inherent anti-plagiarism design in LLM and the ability of LLM to create unique answers. Though raters performed low sensitivity in identifying AI-generated answers, an out-performing gastroenterologist considered that AI-generated answer was more like a structured paragraph. In contrast, answers from public webpages were more like colloquial responses. Our evaluation revealed that AI matched human performance in answering CQs and excelled in conceptual matters on the PRs (RQ1-3). However, AI struggled with questions requiring current domain-specific knowledge, such as treatment plans, prognostic prediction, and follow-up advice (RQ4-6). Despite AI's propensity for generating comprehensible responses, it may contain fabricated or outdated information—a phenomenon known as “hallucination” (15).

We conducted this study as a proof-of-concept and performed thorough quantitative and qualitative analyses to ensure that our findings are statistically rigorous. While the small sample size and specific type of standard PRs in the present study are limitations that may impact the universality of our results, our findings nonetheless reflect the potential of LLMs for improving doctor-patient communication and patients' understanding of complex medical reports. In future works, we plan to expand the sample size to include multi-center data in order to more systematically verify our findings.

As LLMs continue to advance, we have found that more recent models, such as Anthropic's Claude 3 Opus, demonstrate improved readability (Supplementary Table 3) and hold great potential for driving continuous enhancements in the clarity of explanations. In future work, we plan to further develop more advanced LLM-based PRs interpretation systems by integrating external medical data, such as cancer knowledge graphs and clinical guidelines (16). By applying techniques like Retrieval Augmented Generation and tool learning agents, we aim to further enhance the accuracy and response verifiability of LLM-based systems in answering pathology questions (17, 18).

In conclusion, LLMs have shown potential to generate credible answers to common pathology questions. With further enhancements, LLMs hold great promise in improving doctor-patient communication regarding professional PRs.

## Data Availability

The raw data supporting the conclusions of this article will be made available by the authors, without undue reservation.
