# Supplementary material for: Large language model answers medical questions about standard pathology reports
Source: Front Med (Lausanne). 2024 Sep 18;11:1402457. doi: 10.3389/fmed.2024.1402457 (PMC11445125; doi:10.3389/fmed.2024.1402457)
Supplement: Supplementary file 1 [file Data_Sheet_1.docx]

**Information of the generative AI technology used in the study**

Throughout the study, we experimented various large language models and selected Chat Generative Pretrained Transformer or ChatGPT model from OpenAI, Inc. as the main model of our report analysis system. We used the version “gpt-3.5-turbo” at the time of the paper, and as of now this model in our system has been updated to “gpt-3.5-turbo-0125” automatically.

**Prompts used in the study**

We used a predefined system prompt for all the questions evaluated, and for each specific questions we prompted the large language model in our system with the specific user prompt. Our system prompt is as follows,

In English: You are a medical assistant proficient in interpreting colorectal cancer pathology reports. When you answer questions, please combine the context of colorectal cancer. If you do not know how to answer, say you do not know directly, do not talk about things you are unsure of. Do not answer questions unrelated to colorectal cancer. If there are irrelevant questions, respond that you cannot answer such questions. Try to use simple and understandable language, but do not make things up.

In Chinese: 你是一个精通解读结直肠癌病理报告的医学助理，你回答问题的时候请结合结直肠癌这个语境，如果你不知道如何回答，直接说不知道，不要说你不确定的事情。不要回答和结直肠癌无关的问题，如果有无关的问题，回答你无法回答这类问题。尽量用简单易懂的语言，但是不要乱编。

**User prompts are the common questions as listed in supplementary table 1, we do not have other additional prompts.**

**Supplementary Table 1**. Example Answers of Common Questions from AI and Non-AI

| CQs | AI1 answer | | AI2 answer | | Hospital1 answer | | Hospital2 answer | |
| --- | --- | --- | --- | --- | --- | --- | --- | --- |
|  | In English | In Chinese | In English | In Chinese | In English | In Chinese | In English | In Chinese |
| CQ1: What is a pathological examination? | Pathological examination is a method of diagnosing diseases by observing and analyzing changes in tissues and cells. It is an important tool in the field of medicine for determining the nature, progression, and treatment options of diseases. In pathological examinations, doctors typically collect tissue samples from patients, such as biopsies or postoperative tissues. These samples are analyzed through special staining and microscopic observation. Pathologists assess the morphological characteristics of tissues and cells, including cell morphology, tissue structure, cell division activity, and intracellular changes. Through pathological examination, abnormalities in tissues or cells can be identified, such as tumors, inflammation, infections, ischemia, and necrosis. Additionally, pathological examination can determine the type, grade, and stage of tumors, as well as evaluate the prognosis and treatment response of diseases. In summary, pathological examination is an important diagnostic method that provides valuable information about diseases to doctors, helping guide the treatment and management of patients. | 病理学检查是一种通过观察和分析组织和细胞的变化来诊断疾病的方法。它是医学领域中的重要工具，用于确定疾病的性质、进展和治疗方案。在病理学检查中，医生通常会收集患者的组织样本，如活检或切除术后的组织。这些样本会通过特殊的染色和显微镜观察来进行分析。病理学家会评估组织和细胞的形态学特征，包括细胞形态、组织结构、细胞分裂活动和细胞内部的变化。通过病理学检查，可以确定组织或细胞存在的异常，如肿瘤、炎症、感染、缺血、坏死等。此外，病理学检查还可以确定肿瘤的类型、分级和分期，以及评估疾病的预后和治疗反应。总之，病理学检查是一种重要的诊断方法，为医生提供了关于疾病的有价值的信息，帮助指导患者的治疗和管理。 | Pathological examination is a method of examining and analyzing tissues, organs, or cells to aid in the diagnosis and study of diseases. It provides information about the development and nature of diseases by observing and evaluating pathological changes. In pathological examinations, doctors typically collect tissue samples from patients, such as biopsies or surgically removed tissues. After a series of processing and staining, pathologists use microscopes to observe and analyze the samples. They assess the morphology of cells, the structure of tissues, intracellular changes, and any possible abnormalities. Pathological examination can be used to diagnose and differentiate diseases, such as cancer, infectious diseases, and autoimmune diseases. It can also help determine the severity, prognosis, and treatment strategies for diseases. Additionally, pathological examination can be used for scientific research to increase understanding of disease mechanisms and pathophysiology. Overall, pathological examination plays an important role in medical diagnosis and research, providing in-depth insights into disease development and nature, and serving as a crucial basis for guiding the treatment and management of diseases. | 病理学检查是一种通过对组织、器官或细胞进行检查和分析，以帮助诊断和研究疾病的方法。它通过观察和评估病理变化的特征，从而提供有关疾病发展和性质的信息。在病理学检查中，医生通常会收集患者的组织样本，如活检或手术切除的组织。这些样本经过一系列的处理和染色后，病理学家会使用显微镜进行观察和分析。他们会评估细胞的形态、组织的结构、细胞内的变化以及可能存在的异常情况。病理学检查可以用于诊断和鉴别疾病，例如癌症、感染性疾病和自身免疫性疾病。它还可以帮助确定疾病的严重程度、预后以及治疗策略。此外，病理学检查还可以用于科学研究，以增加对疾病机制和病理生理学的理解。总的来说，病理学检查在医学诊断和研究中起着重要的作用，它提供了对疾病发展和性质的深入了解，为医生提供了指导治疗和管理疾病的重要依据。 | Pathological examination refers to the process of obtaining tissue or fluid samples from a specific part of the body, preparing them as pathological sections, and observing them under a microscope to determine the nature of the disease. Pathological examination is the most accurate examination among all clinical tests, with an accuracy rate of about 99%. For example, if there is a mass lesion in the lung, but the nature of the lesion cannot be determined, bronchoscopy or lung puncture biopsy can be performed. By making pathological sections of the lesion tissue, a final diagnosis can be made to determine whether it is a benign or malignant lesion. Pathological examination can also guide treatment, estimate prognosis, assist doctors in selecting surgical treatment plans, and assess the development of the disease and treatment effectiveness. Additionally, performing pathological examination on a corpse helps identify causes and lesions, analyze the relationships between various lesions, diagnose the cause of death, and discover and confirm certain infectious diseases, epidemics, local diseases, and emerging diseases, providing scientific evidence for the implementation of treatment measures by the health authorities. | 病理检查是指将身体某个部位的组织或者体液做成病理切片，在显微镜下观察，通过镜下观察到的形态，判断病变性质。病理检查是临床所有检查项目中最准确的检查，准确率可达到99%左右。比如肺部有占位病变，但是不能确定病变性质，可以做支气管镜或者肺穿刺活检，将占位的组织做成病理切片，可以作出最终诊断，明确是良性还是恶性病变。病理检查还可以指导治疗，估计预后，协助医生选择手术治疗方案，还可以了解治疗过程中的发展情况和判断疗效。另外，对尸体做病理检查，还有助于查出病因、病变，分析各种病变的相互关系，可以诊断死亡的原因，还可以发现和确诊一些传染病、流行病以及地方性疾病、新发疾病，为防疫部门采取治疗措施提供科学依据。 | Pathological examination is a histomorphological method used to examine pathological changes in organs, tissues, or cells. To explore the disease processes occurring in organs, tissues, or cells, a certain method of pathological examination can be employed to investigate the occurring lesions, explore the causes and pathogenic mechanisms of the lesions, and examine the development process of the lesions, ultimately leading to a pathological diagnosis. The examination methods of pathological morphology involve initially observing the pathological changes in gross specimens, then obtaining a certain size of pathological tissue for preparation of pathological sections using histopathological methods, and further examining the lesions under a microscope. Pathological examination has been widely applied in clinical work and scientific research. In clinical practice, it is mainly used for autopsy pathology examination and surgical pathology examination. The purpose of surgical pathology examination is twofold: first, to establish a definitive diagnosis and validate preoperative diagnoses, thereby improving the diagnostic level in clinical settings; second, after a diagnosis is established, it can determine the next step in the treatment plan and estimate the prognosis, thus enhancing the level of clinical treatment. Additionally, a significant amount of valuable scientific research data can be obtained through clinical pathological analysis. | 病理学检查是用以检查器官、组织或细胞中病理改变的病理形态学方法。为探讨器官、组织或细胞所发生的疾病过程，可采用某种病理形态学检查的方法，检查他们所发生的病变，探讨病变产生的原因、发病机理、病变的发生发展过程，最后做出病理诊断。病理形态学的检查方法，首先观察大体标本的病理改变，然后切取一定大小的病变组织，用病理组织学方法制成病理切片，用显微镜进一步检查病变。病理检查已经大量应用于临床工作及科学研究。在临床方面主要进行尸体病理检查及手术病理检查。手术病理检查的目的，一是为了明确诊断及验证术前的诊断，提高临床的诊断水平；二是诊断明确后，可决定下一步治疗方案及估计预后，进而提高临床的治疗水平。通过临床病理分析，也可获得大量极有价值的科研资料。 |

**Supplementary Table 1**. Continued

| CQs | AI1 answer | | AI2 answer | | Hospital1 answer | | Hospital2 answer | |
| --- | --- | --- | --- | --- | --- | --- | --- | --- |
|  | In English | In Chinese | In English | In Chinese | In English | In Chinese | In English | In Chinese |
| CQ2: Why is a pathological examination performed? | Pathological examination is a method of diagnosing diseases by observing and analyzing patient tissues and cells. It plays a crucial role in medical diagnosis and treatment. Here are some key reasons for performing pathological examinations: (1) Disease diagnosis: Pathological examination can help doctors determine whether patients have specific diseases, such as cancer, infectious diseases, autoimmune diseases, etc. By observing and analyzing abnormal changes in tissues and cells, pathologists can provide important information about the type, severity, and prognosis of diseases. (2) Disease monitoring: For diagnosed diseases, pathological examination can be used to monitor the progression of the disease and the effectiveness of treatment. By quantitatively and qualitatively analyzing changes in tissues and cells, it can help doctors evaluate the efficacy of treatment and adjust treatment plans in a timely manner. (3) Surgical guidance: During surgical procedures, pathological examination can provide real-time histological information to help doctors determine the extent and invasion of lesions. This is crucial for precise localization during surgery and determining the scope of the operation, helping to reduce surgical complications and improve the thoroughness of surgical resection. (4) Research and science: Pathological examination is an important tool for medical research and scientific exploration. By observing and analyzing tissues and cells, it can reveal the mechanisms of disease occurrence, pathological and physiological changes, and potential targets for treatment, driving advances in medicine. In summary, pathological examination plays a vital role in disease diagnosis, treatment, and research, providing crucial information and guidance to doctors, thereby helping patients receive better medical care. | 病理学检查是一种通过观察和分析患者组织和细胞的方式，以帮助诊断疾病的方法。它在医学诊断和治疗中起着至关重要的作用。以下是一些做病理学检查的主要原因：（1）疾病诊断：病理学检查可以帮助医生确定患者是否患有特定疾病，例如癌症、感染性疾病、自身免疫性疾病等。通过观察和分析组织和细胞的异常变化，病理学家可以提供有关疾病类型、程度和预后的重要信息；（2）病情监测：对于已经确诊的疾病，病理学检查可以用来监测病情的进展和治疗的效果。通过对组织和细胞的变化进行定量和定性分析，可以帮助医生评估治疗的疗效，并及时调整治疗方案；（3）手术指导：在手术过程中，病理学检查可以提供实时的组织学信息，帮助医生判断病变的范围和侵袭程度。这对于手术的精确定位和决定手术范围非常重要，有助于减少手术并发症和提高手术切除的彻底性；（4）研究和科学：病理学检查是进行医学研究和科学探索的重要工具。通过对组织和细胞的观察和分析，可以揭示疾病的发生机制、病理生理学变化以及治疗的潜在靶点，推动医学的进步。总而言之，病理学检查在疾病诊断、治疗和研究中发挥着重要作用，为医生提供关键的信息和指导，从而帮助患者获得更好的医疗护理。 | Pathological examination plays a crucial role in medical diagnosis and treatment. Here are several key reasons for conducting pathological examinations: (1) Disease diagnosis: Pathological examinations help physicians determine the type and characteristics of diseases. By observing and analyzing tissue, cellular, or fluid samples, pathologists can detect abnormal cells, tumors, inflammations, infections, etc., and determine the nature of the diseases. This is essential for accurate disease diagnosis, treatment planning, and prognosis prediction. (2) Tumor grading and staging: Pathological examinations can assess tumor grading and staging. By observing indicators such as cellular atypia (degree of cellular abnormality), proliferative activity, and depth of infiltration, tumors can be classified into different grades, such as the grading of colorectal cancer. Moreover, by detecting indicators like lymph node metastasis and distant metastasis, tumors can be staged using systems like the TNM staging system. This helps predict disease progression and prognosis, and guides appropriate treatment strategies. (3) Treatment decision-making: Pathological examination results are crucial for developing treatment plans and making decisions. For example, in cancer treatment, pathological examinations can determine the molecular characteristics of tumors, such as HER2 expression, estrogen receptor status, and progesterone receptor status. This information can guide the use of targeted therapies, chemotherapy, or hormone therapy as specific treatment strategies. (4) Monitoring treatment effectiveness: Pathological examinations can evaluate the efficacy of treatments. By analyzing post-treatment tissue or cellular samples, changes in tumors, cellular responses, and treatment effects can be observed and compared. This helps determine if the treatment is effective and if adjustments to the treatment plan are necessary. In summary, pathological examination is an important medical tool that provides in-depth understanding of disease characteristics and nature, offering accurate evidence for diagnosis and treatment. It plays a vital role in personalized medicine, helping physicians develop individualized treatment plans, improve treatment efficacy, and prognosis. | 病理学检查在医学诊断和治疗中起着至关重要的作用。以下是进行病理学检查的几个重要原因：（1）疾病诊断：病理学检查可以帮助医生确定疾病的类型和特征。通过对组织、细胞或体液样本的观察和分析，病理学家可以检测异常细胞、肿瘤、炎症、感染等，并确定疾病的性质。这对于准确诊断疾病、确定治疗方案和预测预后非常重要；（2）病理分级和分期：病理学检查可以评估肿瘤的分级和分期。通过观察肿瘤细胞的异型性（细胞异常程度）、增殖活性和浸润深度等指标，可以将肿瘤分为不同的分级，如结直肠癌的分级。此外，通过检测淋巴结转移和远程转移等指标，可以对肿瘤进行分期，如TNM分期系统。这有助于预测疾病的进展和预后，并指导适当的治疗策略；（3）治疗决策：病理学检查结果对于制定治疗计划和决策非常重要。例如，在癌症治疗中，病理学检查可以确定肿瘤的分子特征，如HER2表达、雌激素受体和孕激素受体状态等。这些信息可以指导是否使用靶向治疗、化疗或激素疗法等特定的治疗策略；（4）观察治疗效果：病理学检查可以评估治疗的效果。通过对治疗后组织或细胞样本的分析，可以观察和比较肿瘤的变化、细胞的反应和治疗效果。这有助于确定治疗是否有效，是否需要调整治疗方案。总的来说，病理学检查是一种重要的医学工具，可以提供对疾病特征和性质的深入了解，为诊断和治疗提供准确的依据。它在个体化医学中起着至关重要的作用，帮助医生制定个性化的治疗方案，提高治疗效果和预后。 | Pathological examination is one of the important methods for diagnosing and treating diseases, and its role is irreplaceable. In pathological examinations, pathologists observe changes at the tissue, cellular, and molecular levels under a microscope to diagnose diseases, evaluate the severity of diseases, and determine the best treatment plans. For example, in tumor pathology examinations, pathologists can identify tumor types, grade and stage tumors, and provide important basis for developing optimal treatment plans for tumor patients. At the same time, pathological examinations can also help differentiate between various diseases, such as the difference between cancer and benign tumors. Pathological examinations also support medical research and drug development. Through pathological examinations, the pathogenesis and pathophysiological processes of diseases can be understood, providing important scientific basis for the treatment and prevention of diseases. Pathological examinations can also evaluate the safety and efficacy of new drugs, providing support for drug development. In addition, pathologists can verify and develop new treatment methods by detecting tissue and cellular markers. Pathological examinations can also provide early warning and screening for diseases. For example, histological examination of colorectal cancer can detect early signs of carcinoma, providing possibilities for early detection and treatment. In addition, pathological examinations can provide early warning for genetic diseases, enabling early detection of potential risks and preparing for genetic counseling and intervention. Furthermore, pathological examinations can even aid in historical research, such as revealing diseases and causes of ancient human remains. Pathological examination is crucial for confirming or treating diseases, evaluating treatment effects, and participating in research on drugs and treatment methods. | 病理检查是病理检查是诊断和治疗疾病的重要手段之一，其作用不可替代。在病理学检查中，病理学家会通过显微镜观察组织、细胞和分子层面的变化，从而诊断疾病、评估疾病的严重程度和确定最佳治疗方案。例如，在肿瘤病理学检查中，病理学家可以确定肿瘤类型、分级和分期，为肿瘤患者制定最佳的治疗计划提供重要依据。同时病理检查也可以帮助鉴别各种疾病之间的差异，例如癌症和良性肿瘤之间的区别。病理检查还可以为医学研究和新药开发提供支持。通过病理检查，可以了解疾病的发病机制和病理生理学过程，为疾病的治疗和预防提供重要的科学依据。病理检查还可以评估新药的安全性和有效性，为药品的研发提供支持。此外，病理学家还可以通过检测组织和细胞标记物来验证和开发新的治疗方法。病理检查还可以对疾病进行预警和筛查。例如，通过对大肠癌的组织学检查可以检测到早期癌变的迹象，为早期发现和治疗提供了可能性。此外，病理检查还可以对遗传性疾病进行预警，及早发现可能存在的风险，为遗传咨询和干预提前做好准备。此外，病理学检查甚至可以帮助历史研究，如揭示古人类遗骸的疾病和死因。对于确诊或治疗疾病，评估治疗效果，参与药物和治疗方法的研究，病理学检查都是至关重要的。 | There are several reasons for performing pathological examinations: (1) Pathological examinations enable timely and accurate diagnoses of diseases, guiding treatment decisions and estimating prognosis for patients. (2) Pathological examinations help identify the nature of lesions before surgery, enabling the issuance of diagnostic reports and assisting clinicians in selecting appropriate surgical treatment plans. (3) Pathological examinations provide insights into the development of diseases and help assess the efficacy of treatments during the observation and treatment processes. (4) By utilizing innovative pathological examination methods, a more in-depth study of diseases becomes possible. Additionally, conducting pathological examinations on deceased individuals facilitates the identification of causes and lesions, analysis of the primary and secondary relationships between various lesions, determination of diagnoses, and determination of the cause of death. It also enables the timely detection and confirmation of certain infectious diseases, endemic diseases, epidemics, and newly emerging diseases, providing a basis for the implementation of treatment measures by public health authorities. | 因为以下原因要做病理检查：第一，病理检查可以及时准确的对患者作出疾病的病理诊断，并指导治疗，估计预后。第二，病理检查可以为患者手术时明确病变性质，发出诊断报告，协助临床医生选择手术治疗方案。第三，可以在疾病观察和治疗过程中，了解病变发展情况或判断疗效。第四，采用一些新的病理检查方法，能够对疾病进行更深入的研究。另外，对于尸体做病理检查，有利于查出病因、病变，分析各种病变的主次和相互关系，确定诊断，查明死因。还可以及时发现和确诊某些传染病、地方病、流行病和新发生的疾病，为防疫部门采取治疗措施提供依据。 |

**Supplementary Table 1**. Continued

| CQs | AI1 answer | | AI2 answer | | Hospital1 answer | | Hospital2 answer | |
| --- | --- | --- | --- | --- | --- | --- | --- | --- |
|  | In English | In Chinese | In English | In Chinese | In English | In Chinese | In English | In Chinese |
| CQ3: What to constitute a pathological report? | The pathology report is written by a pathologist and contains a detailed description of the pathological examination results and diagnostic conclusions. The specific components of a pathology report may vary depending on the preferences of different laboratories and pathologists, but generally include the following main sections: (1) Specimen information: This includes basic patient information such as name, age, gender, as well as the source and collection date of the specimen. This information helps ensure that the report is associated with the correct patient and pathological sample. (2) Clinical information: This section includes the patient's medical history, chief complaints, clinical manifestations, etc. Clinical information is crucial for interpreting and diagnosing the pathological results because it provides background and contextual information. (3) Specimen description: The pathologist provides a detailed description of the received specimen, including its size, shape, color, and any significant features. Additionally, the collection and processing methods of the specimen are also recorded. (4) Histological description: This section provides a detailed description of the histological features. The pathologist observes the morphological characteristics of tissue or cell samples and describes any abnormal changes. For example, in the case of tumor specimens, the type, grade, depth of invasion, and cellular characteristics of the tumor would be described. (5) Diagnostic conclusion: Based on the observation and analysis of the specimen, the pathologist provides a diagnostic conclusion. This may include a specific diagnosis of the disease, classification and grading of the tumor, severity of the lesion, etc. The diagnostic conclusion is typically the core content of the entire pathology report. (6) Additional information: Depending on the specific circumstances, the pathology report may include additional information such as special staining results, immunohistochemical test results, molecular genetic analysis results, etc. These pieces of information can provide more detailed diagnostic and prognostic information. It should be noted that the content and format of a pathology report may vary depending on the requirements of the laboratory and hospital. Some pathology reports may also include supplementary information such as images, charts, etc., to better support the pathologist's views and diagnosis. | 病理报告是由病理学家编写的，它包含了对病理学检查结果的详细描述和诊断结论。病理报告的具体组成可以根据不同的实验室和病理学家的偏好而有所不同，但通常包括以下几个主要部分：（1）标本信息：包括病人的基本信息，如姓名、年龄、性别，以及标本的来源和采集日期。这些信息有助于确保报告与正确的患者和病理样本相关联；（2）临床信息：这一部分包括患者的病史、主诉、临床表现等。临床信息对于与病理学结果的解释和诊断非常重要，因为它可以提供背景和上下文信息；（3）标本描述：病理学家会对接收到的标本进行详细的描述。这包括标本的大小、形状、颜色以及任何显著的特征。此外，标本的采集方式和处理方法也会被记录下来；（4）组织学描述：这一部分是对组织学特征的详细描述。病理学家会观察组织或细胞样本的形态学特征，并描述异常变化。例如，对于肿瘤标本，会描述肿瘤的类型、分级、浸润深度、细胞特征等；（5）诊断结论：基于对标本的观察和分析，病理学家会给出诊断结论。这可能是对疾病的具体诊断、肿瘤的分类和分级、病变的程度等。诊断结论通常是整个病理报告的核心内容；（6）其他附加信息：根据具体情况，病理报告还可能包括其他附加信息，如特殊染色结果、免疫组化检测结果、分子遗传学分析结果等。这些信息可以提供更详细的诊断和预后信息。需要注意的是，病理报告的内容和格式可能会因实验室和医院的要求而有所不同。有些病理报告可能还包括图片、图表等辅助信息，以更好地支持病理学家的观点和诊断。 | Pathology reports typically consist of several sections: (1) Patient Information, including the patient's name, age, gender, hospital ID, and other basic details that help uniquely identify and track the patient; (2) Specimen Description, which describes the source and characteristics of the tissue specimen submitted for examination, such as surgical resection or biopsy specimens; (3) Specimen Processing, detailing the laboratory procedures involved in handling the specimen, including fixation, sectioning, staining, and other technical details; (4) Histological Description, providing a description of the tissue structure and cell types observed in the specimen, such as tumor type, grading, depth of invasion, etc.; (5) Pathological Diagnosis, the final diagnosis based on the histological description and clinical information, indicating whether it is a benign tumor, malignant tumor, inflammation, etc.; (6) Immunohistochemistry Results, if immunohistochemical staining is performed, the report will list the antibodies used and the staining results; (7) Molecular Pathology Results, if molecular pathology testing is conducted, the report will include the testing methods and results, such as gene mutations, fusion genes, etc.; (8) Diagnostic Opinion and Recommendations, providing treatment suggestions or further investigations based on the pathological diagnosis. The specific content of a pathology report may vary depending on the type of tissue specimen and clinical circumstances, but these sections are typically included in the report. | 病理报告通常由以下几个部分组成：（1）病例信息：包括患者姓名、年龄、性别、住院号等基本信息，这些信息有助于唯一标识和追踪患者；（2）标本描述：描述送检的组织标本的来源和特征，例如手术切除标本、活检标本等；（3）标本处理：描述标本在实验室中的处理过程，包括固定、切片、染色等技术细节；（4）组织学描述：对标本中的组织结构和细胞类型进行描述，例如肿瘤的类型、分级、浸润深度等；（5）病理诊断：根据组织学描述和临床信息，给出最终的病理诊断，例如良性肿瘤、恶性肿瘤、炎症等；（6）免疫组化结果：如果进行了免疫组化染色检查，会在报告中列出所用抗体和染色结果；（7）分子病理学结果：如果进行了分子病理学检查，会在报告中列出检测方法和结果，例如基因突变、融合基因等；（8）诊断意见和建议：根据病理诊断，提供对患者治疗或进一步检查的建议。病理报告的具体内容会根据不同类型的组织标本和临床情况而有所差异，但以上部分通常都会包含在报告中。 | Composition of Pathology Report: (1) Patient Information and Submission Information: including the patient's name, gender, age, pathology number, submitting department, submitted specimen, submission date, etc. The pathology number is a unique identifier for each patient's specimen block and pathology slides. Patient-related information can be directly extracted by the pathology department using the pathology number. (2) Macroscopic Observation of Pathological Specimens: primarily describing the size, shape, color, presence of tumors, as well as key information such as the size, color, morphology, consistency, and distance from the margin of the tumor. It should also indicate the method and site of specimen acquisition, such as biopsy, endoscopy, or surgical excision. (3) Microscopic Observation: The pathologist provides a professional description of the histological structure and cytological changes observed under the microscope after staining the specimen slides, including immunohistochemistry (IHC) or special staining results. (4) Pathological Diagnosis: This is the most important part of the pathology report. In this section, the pathologist provides a professional diagnosis, including whether it is a tumor, whether the tumor is benign or malignant. If malignant, it is necessary to determine the size, degree of differentiation, depth of invasion, presence of lymph node metastasis, margin status, presence of vascular emboli and nerve invasion, and pathological staging. This information serves as an important reference for clinical treatment. | 病理报告的组成：（1）患者信息和送检信息：包括患者的姓名、性别、年龄、病理号、送检科室、送检标本、送检日期等。其中，病理号是每个患者的标本蜡块以及病理切片的唯一号码，在病理科通过病理号就可直接提取患者相关信息；（2）病理标本的肉眼观察：主要描述标本的大小、形状、颜色、有无肿瘤以及肿瘤的大小、颜色、形态、质地、距切缘距离等关键信息。同时也要注明送检标本来源的方式和部位，即标本来源于哪个器官，通过哪种方式获取的，如穿刺、腔镜或者手术切除等；（3）描述显微镜下观察到的内容：病理医师对标本切片染色后在显微镜下观察到的组织学结构及细胞形态学改变进行专业性的描述，包括免疫组织化学染色（免疫组化）或特殊染色等结果；（4）病理诊断：这是病理报告中最重要的部分，在这一部分，病理医生会给出一个专业性的诊断，包括是否为肿瘤，肿瘤是良性还是恶性，如果是恶性的，则需要确定该恶性肿瘤的大小、分化程度、浸润深度、有无淋巴结转移，切缘是否干净、有无脉管癌栓和神经侵犯以及病理分期等信息，这些信息会作为临床治疗的重要参考依据。 | The pathological report consists of the following parts: (1) Patient information and specimen information, including the patient's name, gender, age, pathology number, medical record number, department of reception, and date of specimen submission. The pathology number is a unique code for each patient's specimen block and pathological slides. It allows the pathology department to directly retrieve relevant patient information based on the pathology number. (2) Macroscopic description of the submitted specimen, which describes the size, shape, color, presence of tumors, as well as key information about the size, color, morphology, texture, and distance from the surgical margin of the tumor. (3) Microscopic observations, i.e., the histopathological findings under the microscope. Generally, the term "large specimen" refers to the specimen obtained after surgical resection, while "small specimen" refers to specimens obtained through bronchoscopy, endoscopy, gastroscopy, or superficial biopsies. If it is a small specimen, the source organ or tissue and the method of acquisition are usually described first. Then, a professional description of the histological structure and cytological changes observed under the microscope is provided. (4) Pathological diagnosis: This is the key part to focus on when reading the pathological report. In this section, the pathologist provides a professional diagnosis, including whether it is a tumor, whether the tumor is benign or malignant. If it is malignant, the pathologist will determine the severity of the malignant tumor based on the observations of the slides. This includes the size of the tumor, degree of differentiation, depth of invasion, presence of lymph node metastasis, status of surgical margins, presence of vascular invasion and nerve infiltration, as well as pathological staging. Sometimes, there may also be immunohistochemistry results below the diagnosis, which can assist in diagnosis or guide treatment. | 病理报告由以下部分组成：（1）患者信息和送检信息：包括患者的姓名、性别、年龄、病理号、病案号、接诊的科室、送检日期等。其中，病理号是每个患者的标本蜡块以及病理切片的唯一号码，在病理科通过病理号就可直接提取病人相关信息；（2）即送检标本宏观大体上长什么样子，它描述了切除标本的大小、形状、颜色、有无肿瘤以及肿瘤的大小、颜色、形态、质地、距切缘距离等关键信息；（3）显微镜下观察到的结果，即镜下所见：一般来说，病理医生所说的大标本是指手术切除后获取的标本，小标本则是指通过支气管镜、腔镜、胃镜获取的或者体表活检获取的标本称为小标本。如果是小标本，镜下所见一般会首先描述标本来源于哪种器官或组织以及通过哪种方式获取的。其次，会对显微镜下所观察到的组织学结构及细胞形态学改变进行比较专业性的描述；（4）病理诊断：这是看病理报告时要重点关注的内容，在这一部分，病理医生会给出一个专业性的诊断，包括是否为肿瘤，肿瘤是良性还是恶性，如果是恶性的，病理科医生会根据对切片的观察，确定该恶性肿瘤的严重程度，包括肿瘤的大小，分化程度、浸润深度、有无淋巴结转移，切缘是否干净、有无脉管癌栓和神经侵犯以及病理分期。有时诊断下面还会有免疫组化结果，免疫组化可以辅助诊断或指导治疗。 |

**Supplementary Table 1**. Continued

| CQs | AI1 answer | | AI2 answer | | Hospital1 answer | | Hospital2 answer | |
| --- | --- | --- | --- | --- | --- | --- | --- | --- |
|  | In English | In Chinese | In English | In Chinese | In English | In Chinese | In English | In Chinese |
| CQ4: What is an immunohistochemical result? | Immunohistochemistry is a commonly used pathological technique for detecting the presence and localization of specific proteins in tissue samples. The immunohistochemical results refer to the expression of specific proteins observed in tissue during the immunohistochemistry experiment. Immunohistochemical techniques rely on the specific binding between antibodies and specific antigens. First, the specimen tissue is sliced and fixed on slides. Then, the fixed tissue is stained with antibodies specific to the target antigen. If the target protein is present in the tissue sample, the antibody will bind to it, forming an immune complex. Finally, the immune complex is labeled with visible dyes or fluorescent dyes to make it visible under a microscope. Immunohistochemical results can provide information about the localization, expression levels, and distribution patterns of the target protein in the tissue. This is crucial for the diagnosis and classification of diseases. For example, in tumor pathology, immunohistochemistry can help determine the origin, grade, and molecular subtype of the tumor. It can also be used in clinical practice to determine treatment plans, such as assessing whether a tumor is sensitive to specific immunotherapy drugs. Immunohistochemistry is typically observed and interpreted under a microscope. By evaluating the intensity, distribution, and localization of the staining, pathologists can interpret immunohistochemical results and compare them with standards or reference ranges to reach a diagnostic conclusion. It is important to note that immunohistochemical results are only part of pathological assessment and require comprehensive analysis and diagnosis in conjunction with clinical information, histological features, and other test results. | 免疫组化是一种常用的病理学技术，用于检测组织样本中特定蛋白质的存在和定位。免疫组化结果指的是在免疫组化实验中观察到的特定蛋白质在组织中的表达情况。免疫组化技术基于抗体与特定抗原的特异性结合。首先，标本组织被切片并固定在载玻片上。然后，对固定的组织进行特定抗原的抗体染色。如果目标蛋白质存在于组织样本中，抗体将与其结合形成免疫复合物。最后，通过可见的染色剂或荧光染料，将免疫复合物标记出来，使其在显微镜下可见。免疫组化结果可以提供有关目标蛋白质在组织中的定位、表达水平和分布模式的信息。这对于疾病的诊断和分类非常重要。例如，在肿瘤病理学中，免疫组化可以帮助确定肿瘤的起源、分级和分子亚型。它还可以在临床实践中用于确定治疗方案，例如判断肿瘤是否对特定的免疫治疗药物敏感。免疫组化通常使用显微镜观察和解读。通过评估染色的强度、分布和定位，病理学家可以解读免疫组化结果，并将其与标准或参考范围进行比较，从而得出诊断结论。需要注意的是，免疫组化结果只是病理学评估的一部分，需要结合临床信息、组织学特征和其他检查结果来进行综合分析和诊断。 | Immunohistochemistry is a commonly used histological detection technique used to determine the expression of specific proteins in tissues. Immunohistochemistry results refer to the staining results obtained in immunohistochemical testing. The immunohistochemistry technique is based on the specific binding between antibodies and specific antigens. By using specific antibodies, the presence and expression levels of target proteins in tissues can be detected. The technique mainly involves the following steps: tissue fixation, antigen retrieval, antibody staining, observation of color development, and staining reaction. In immunohistochemistry results, dyes such as molybdenum dioxide (MoO2) and DAB (3,3'-diaminobenzidine) are commonly used to visualize the areas where the target protein is expressed, presenting distinct colors. A positive result indicates the presence of the target protein in the tissue, and the intensity of staining reflects the level of its expression. A negative result indicates that the target protein was not detected in the tissue or its expression level is very low. Immunohistochemistry results are of significant importance in pathological diagnosis. They can be used to determine tumor types, assess tumor grading, evaluate prognostic indicators, and guide treatment selection. Immunohistochemistry results are typically listed in pathology reports for reference by clinical doctors. | 免疫组化是一种常用的组织学检测技术，用于检测组织中特定蛋白质的表达情况。免疫组化结果指的是在免疫组化检测中得到的染色结果。免疫组化技术基于抗体与特定抗原之间的特异性结合。通过使用特定抗体，可以检测组织中的目标蛋白质是否存在以及其表达水平。该技术主要包括以下步骤：固定组织切片、抗原修复、抗体染色、显色和染色反应的观察。在免疫组化结果中，通常使用染色剂（如二氧化钼、DAB）使目标蛋白质表达的区域呈现出明显的颜色。阳性结果表明目标蛋白质在组织中存在，并且染色强度的程度可以反映其表达水平的高低。阴性结果表示目标蛋白质在组织中未被检测到或表达水平非常低。免疫组化结果在病理诊断中具有重要意义。它可以用于确定肿瘤类型、判断肿瘤的分级、评估预后指标、指导治疗选择等。免疫组化结果通常在病理报告中列出，以提供临床医生参考。 | Immunohistochemistry (IHC) is a technique that utilizes the principle of specific binding between immunological antigens and antibodies. It involves the use of labeled antibodies coupled with colorimetric agents (such as fluorophores, enzymes, metal ions, or isotopes) to visualize the presence of antigens (peptides and proteins) within tissues or cells. This technique allows for the localization, qualitative assessment, and relative quantification of the antigens. | 免疫组化检查指应用免疫学抗原与抗体特异性结合的原理，通过化学反应使标记抗体的显色剂（荧光素、酶、金属离子、同位素）显色，确定组织或细胞内抗原（多肽和蛋白质），对其进行定位、定性及相对的定量检查。 | Immunohistochemistry is a drug detection technique that uses antibodies or markers to localize specific sites on cells or tissues based on their inherent, synthetic, and regulatory activities. It is used to identify and quantitatively analyze cells antigens, cell membrane antigens, variant antigens, epigenetic modifications, and other activities through the immunosignaling system. Immunohistochemistry can deeply identify specific cells or reveal differences between cells, such as the location, quantity, and structure of antigen expression. It can be used in cellular molecular biology research and early detection and subtype classification of pathological tissues in disease diagnosis. Immunohistochemistry techniques can also be used in drug development, where known target sites and signaling pathways are identified and analyzed to improve the efficiency and accuracy of drug development, based on drug targets. | 免疫组化是一种药物检测技术，根据细胞或组织的活动内在，合成和调节的活性，将抗体或标记定位到细胞或组织的特定位点，以识别和通过免疫信号传导系统对其进行定量分析。免疫组化常用于检测细胞抗原、细胞膜抗原、变异抗原、表观遗传修饰以及其他活性。它可以深入的鉴定某种特定的细胞或者揭示细胞之间的不同，如：抗原表达的位置、数量、结构等，可以用于细胞分子的细胞生物学研究，以及疾病诊断中对病变组织的早期检测和子类型分类。免疫组化技术也可以用于药物研发，即以药物靶点为基础，开发新型药物，从而识别和分析已知靶位点和信号通路，以提高药物研发的效率和精确性。 |

**Information of the generative AI technology used in the study**

Throughout the study, we experimented various large language models and selected Chat Generative Pretrained Transformer or ChatGPT model from OpenAI, Inc. as the main model of our report analysis system. We used the version “gpt-3.5-turbo” at the time of the paper, and as of now this model in our system has been updated to “gpt-3.5-turbo-0125” automatically.

Prompts used in the study

We used a predefined system prompt for all the questions evaluated, and for each specific common questions we prompted the large language model in our system with the specific user prompt Our system prompt is as follows,

In English: You are a medical assistant proficient in interpreting colorectal cancer pathology reports. You will explain the contents of the report to patients using professional language. The full text of the report is enclosed within triple backticks ```. When answering questions, please do so in the context of colorectal cancer. If you don't know how to answer, say so directly, without speculating on uncertainties. Pay attention to numerical parts that may have lost the multiplication sign due to OCR recognition; use your judgment in context, especially regarding volume and area. For example, the size 3.00.80.6cm refers to 3.0x0.8x0.6cm. Please determine this on your own, and correct the numerical symbols when responding. Do not answer questions unrelated to colorectal cancer. If there are irrelevant questions, respond that you are unable to answer them. Below is the full text of the report '''{full_text}'''. Please introduce the main content of the report in less than 200 words, including gross findings, microscopic findings, and pathological diagnosis, using simple language that is easy to understand, but do not fabricate details. If you know, describe the possible diagnostic methods and prognostic outcomes for this pathological grade.

In Chinese: 你是一个精通解读结直肠癌病理报告的医学助理，你会用专业的语言向患者解释报告中的内容，报告全文在``` ```中。你回答问题的时候请结合结直肠癌这个语境，如果你不知道如何回答，直接说不知道，不要说你不确定的事情。注意其中的数字部分可能在OCR识别过程中丢失*乘法符号，请你结合语境自己判断，尤其是体积和面积。比如大小3.00.80.6cm指的是3.0x0.8x0.6cm,请你自己判断，在回答的过程中输出你处理过的数字符号。不要回答和结直肠癌无关的问题，如果有无关的问题，回答你无法回答这类问题。以下是报告的全文 '''{full_text}'''，请用200字以内，介绍一下报告的主要内容，包括肉眼所见、镜下所见、病理诊断等部分，尽量用简单易懂的语言，但是不要乱编，如果你知道，描述一下针对这个病理分级，可能的诊断手段和预后效果。

Our user prompt for each question are as follows.

| **Question** | **User Prompt** | |
| --- | --- | --- |
|  | **In English** | **In Chinese** |
| RQ1: Summary of pathological type of tumor. | Summarize the type of tumor in this pathology report. | 总结这份病理报告的肿瘤类型 |
| RQ2: Interpretation of pathological stage. | Explain the pathological staging described in this report. | 解释这份报告的病理分期 |
| RQ3: Interpretation of immunohistochemical results. | Interpret the immunohistochemistry and genetic molecular findings of this report. | 解释这份报告的免疫组化和基因分子结果 |
| RQ4: Interpretation of immunohistochemical results. | How to choose adjuvant therapy after surgery based on this pathology report. | 根据这份病理报告如何选择术后辅助治疗 |
| RQ5: Prediction of prognosis. | Given this pathology report, what is the prognosis for the patient? | 针对这份病理报告, 患者的预后怎么样 |
| RQ6: Advice for follow-up. | Based on this pathology report, provide some suggestions for postoperative follow-up. | 结合这份病理报告, 提出术后随访的一些建议 |

**Supplementary Table 2**. Example Answers of Report Questions from AI and Non-AI

| RQs | User Prompt | | AI answer | | Non-AI answer | |
| --- | --- | --- | --- | --- | --- | --- |
|  | In English | In Chinese | In English | In Chinese | In English | In Chinese |
| RQ1: Summary of pathological type of tumor. |  |  | According to the pathological report, the pathological type of this tumor is moderately differentiated adenocarcinoma. Moderately differentiated adenocarcinoma is a common type of colorectal cancer, formed by malignant proliferation of intestinal glandular epithelial cells. The cellular structure and functional characteristics of moderately differentiated adenocarcinoma are similar to normal colorectal glandular cells, but there are certain abnormal cellular features that lead to increased malignant behavior and metastatic ability. | 根据病理报告，这个肿瘤的病理类型是中分化腺癌。中分化腺癌是一种常见的结直肠癌类型，由肠道腺上皮细胞恶性增生形成。中分化腺癌的细胞结构和功能特征类似于正常的结直肠腺体细胞，但存在某些异常的细胞特点，导致其恶性行为和扩散能力增加。 | The pathological type of the tumor in this patient is moderately differentiated adenocarcinoma. | 该患者肿瘤的病理类型是中分化腺癌。 |
| RQ2: Interpretation of pathological stage. | Explain the pathological staging of this report. | 解释这份报告的病理分期。 | According to the pathological report, the pathological stage of this patient is pT3N0M0. In this report, pT3 indicates that the tumor has invaded the entire layers of the colon wall, including the mucosa and muscularis propria, reaching the serosa. This means that the tumor has started to spread to the surrounding tissues of the colon. N0 indicates no lymph node metastasis, indicating that pathological examination did not find tumor cells spreading to nearby lymph nodes. M0 indicates no distant metastasis. In summary, based on the pathological staging of this report, the patient is staged as pT3N0M0, indicating that the tumor has spread to the entire layers of the colon wall and no lymph node or distant metastasis has been found. | 根据病理报告，该患者的病理分期为pT3N0M0。在这份报告中，pT3表示肿瘤侵犯了结直肠壁的全层，包括内膜、肌层，达到外膜层。这意味着肿瘤已经开始扩散到结直肠周围的组织。N0表示未发现淋巴结转移，表示病理检查未发现肿瘤细胞在邻近淋巴结中的扩散。M0表示无远处转移。综上所述，根据这份报告的病理分期，该患者被分期为pT3N0M0，表示肿瘤已扩散到结直肠壁全层并未发现淋巴结及远处转移。 | According to the 8th edition of AJCC staging criteria, the pathological staging of this patient is T3N0M0 stage, stage IIA. | 根据AJCC第八版分期标准，该患者的病理分期为T3N0M0期，IIA期。 |

**Supplementary Table 2**. Continued

| RQs | AI answer | | Non-AI answer | |
| --- | --- | --- | --- | --- |
|  | In English | In Chinese | In English | In Chinese |
| RQ3: Interpretation of immunohistochemical results. | According to the pathological report, the immunohistochemical results showed positive and negative expressions of several markers. The markers with positive expression include CK20, CDX2, Villin, SATB2, MSH2, MSH6, MLH1, PMS2, and TopoII. The positive expression of these markers indicates normal or enhanced protein expression in tumor cells. CK20 and CDX2 are specific markers for colon cells, and their positive expression further supports the diagnosis of colon cancer. The positive expression of Villin, SATB2, and MUC2 also corresponds to the diagnosis of colon adenocarcinoma. MSH2, MSH6, MLH1, and PMS2 are DNA mismatch repair-related proteins, and their positive expression helps exclude hereditary non-polyposis colorectal cancer syndrome. The positive expression of a few MUC2 and weak focal MUC5-AC suggests the presence of partial mucin secretion within the tumor. The positive expression of P53 and Ki67 reflects the proliferation and apoptosis of tumor cells. P53 mutation accounts for approximately 80%, and Ki67 positivity accounts for approximately 90%. These results indicate the strong proliferative ability of tumor cells. In conclusion, based on the immunohistochemical results in this pathological report, the expression pattern of tumor cells suggests features of colon cancer, with abnormal expression of some markers. These immunohistochemical results help to accurately determine the tumor type and characteristics, assisting doctors in formulating more appropriate treatment plans. | 根据病理报告，免疫组化结果显示了多个指标的阳性和阴性表达。阳性表达的指标包括：CK20、CDX2、Villin、SATB2、MSH2、MSH6、MLH1、PMS2和TopoII。这些标记物的阳性表达表明肿瘤细胞对这些蛋白质的表达呈现正常或增强的情况。CK20和CDX2是结直肠细胞特异性标记物，其阳性表达进一步支持了该肿瘤为结直肠癌。Villin、SATB2和MUC2的阳性表达也与结直肠腺癌的诊断相符。MSH2、MSH6、MLH1和PMS2是DNA错配修复相关蛋白，其阳性表达有助于排除遗传性非聚氨酯瘤综合征。少数MUC2和局灶弱的MUC5-AC的阳性表达表明肿瘤内部可能存在部分粘液分泌。P53和 Ki67的阳性表达反映了肿瘤细胞的增殖和凋亡情况。P53突变型约占80%，Ki67阳性约占90%。这些结果提示了肿瘤细胞的较强增殖能力。综上所述，根据这份病理报告中的免疫组化结果，肿瘤细胞的表达模式表明其具有结直肠癌的特征，并且存在一些异常标记物的异常表达情况。这些免疫组化结果有助于更准确地确定肿瘤类型和特征，帮助医生制定更合适的治疗方案。 | The interpretation of the immunohistochemistry results for this patient is as follows:(1) CD20 is a cytokeratin expressed in intestinal epithelial cells, and its positivity generally serves as evidence that the tumor originates from the intestine. (2) CDX2 is a transcription factor that is specifically expressed in the intestine. There is evidence suggesting that CDX2 positivity (high expression) can be considered as a good prognostic biomarker. (3) Villin is a binding protein of calcium-regulated myosin and is specifically expressed in digestive tract adenocarcinoma tissues. Its positivity can also serve as evidence that the tumor originates from the intestine. (4) SATB2 is also one of the specific markers of colorectal cancer. However, its sensitivity is not as high as Villin and CDX2. It is generally used in combination with other markers to improve diagnostic accuracy. (5) MUC2 is a member of mucin proteins. Decreased expression is a poor prognostic predictor. Some studies suggest that the choice of adjuvant chemotherapy for stage II-III colorectal cancer should consider the expression of MUC2. (6) P53 is a tumor suppressor gene, and mutations in it are one of the most common causes of cancer progression. High expression of mutant P53 indicates poor tumor differentiation, high malignancy, and poor prognosis for patients. (7) Ki67 is an important protein in cell division. High expression of Ki67 is considered a poor prognostic indicator for colorectal cancer patients. (8) D2-40 is a specific marker of lymphatic endothelial cells. It is an important indicator for assessing lymphatic invasion. Positivity in blood vessels suggests that the patient may have lymphatic extension or invasion, indicating a poorer prognosis with a tendency for lymphatic metastasis. (9) MSH2, MSH6, MLH1, and PMS2 are important components of mismatch repair proteins. Positivity for all four proteins indicates that the patient has a microsatellite stable phenotype, which can be used to differentiate hereditary colorectal cancer and provide a basis for immunotherapy. (10) MUC5-AC is a member of mucin proteins. Its upregulation in colorectal cancer indicates enhanced proliferation, invasion, migration capability of tumor cells, as well as increased resistance to certain chemotherapy drugs. (11) TOPOII is topoisomerase II. A higher positivity rate indicates effectiveness against anthracycline antibiotics and podophyllotoxin drugs, with particularly high effectiveness against VP-16. | 该患者的免疫组化结果解读如下：（1）CD20是肠上皮细胞中表达的细胞角蛋白，阳性一般作为肿瘤来源于肠道的证据；（2）CDX2是一种肠特异性表达的转录因子，有证据表明CDX2阳性（高表达）可视为预后良好的生物标志物；（3）Villin是钙调节性肌动蛋白的结合蛋白，在消化道腺癌组织中特异性表达，阳性也可作为肿瘤来源于肠道的证据；（4）SATB2也是结直肠癌的特异性标志物之一，然而敏感性不如Villin和CDX2，一般作为套餐联用提高诊断的准确度；（5）MUC2是粘蛋白中的一员，表达下降是预后不良的预测因素，有研究认为II-III期结直肠癌的辅助化疗选择应考虑MUC2表达情况；（6）P53是一种肿瘤抑制因子，突变型是导致癌症进展的最常见的诱因之一，突变型P53高表达象征肿瘤分化程度差、恶性程度高、患者预后不良；（7）Ki67是细胞分裂中的重要蛋白，Ki67的高表达被视作结直肠癌患者预后的不良的指标；（8）D2-40是淋巴管内皮细胞的特异性标记物，是判断淋巴管侵犯的重要标志，脉管阳性说明该患者可能存在淋巴管扩展或侵犯，肿瘤具有淋巴管转移的趋势，提示预后较差；（9）MSH2、MSH6、MLH1、PMS2是错配修复蛋白的重要组成蛋白，4种蛋白均为阳性提示患者为微卫星稳定型，可用于遗传性结直肠癌的鉴别以及为免疫治疗提供依据；（10）MUC5-AC是粘蛋白中的一员，在结直肠癌中表达上调，阳性表达可能预示肿瘤细胞的增殖、侵袭、迁移能力增强以及对某些化疗药的耐药性增强；（11）TOPOII为拓扑异构酶II，阳性率越高预示着对蒽环类抗生素和鬼臼毒素类药物有效，阳性率高者对VP-16尤其有效。 |

**Supplementary Table 2**. Continued

| RQs | AI answer | | Non-AI answer | |
| --- | --- | --- | --- | --- |
|  | In English | In Chinese | In English | In Chinese |
| RQ4: Interpretation of immunohistochemical results. | Based on the pathological staging and immunohistochemistry results in the pathological report, several aspects need to be considered for the postoperative adjuvant treatment of this patient: (1) Pathological staging: The pathological staging of this patient is pT3N0M0, indicating that the tumor has invaded the full thickness of the colon wall but no lymph node metastasis or distant metastasis has been found. Based on the current pathological staging, routine postoperative adjuvant chemotherapy is not recommended. (2) Immunohistochemistry results: The immunohistochemistry results reveal certain characteristics of the tumor cells, such as positive expression of CK20 and CDX2, which are consistent with the diagnosis of colorectal cancer. This further supports the recommendation against routine chemotherapy. (3) Overall condition of the patient: In addition to the results in the pathological report, the doctor needs to consider the patient's age, physical condition, comorbidities, and the extent of surgical resection. These factors will also influence the choice of postoperative adjuvant treatment. Ultimately, the decision regarding the postoperative adjuvant treatment plan should be made through multi-disciplinary team discussion, taking into account the patient's individual characteristics. It is advised that the patient have further discussions and consultations with the attending physician to jointly develop the most suitable treatment plan. | 根据病理报告中的病理分期和免疫组化结果，对于该患者的术后辅助治疗，以下几个方面需要考虑：（1）病理分期：该患者的病理分期为pT3N0M0，表示肿瘤已经扩散到结直肠壁全层，但未发现淋巴结转移和远处转移。根据目前的病理分期，不推荐常规的术后辅助化疗；（2）免疫组化结果：免疫组化结果显示了肿瘤细胞的一些特点，例如CK20和CDX2阳性表达与结直肠癌的诊断相符。这一结果进一步支持了无需常规的化疗方案；（3）患者的整体情况：除了病理报告的结果，医生还需要综合考虑患者的年龄、身体状况、合并疾病、手术的切除范围等因素。这些因素也会影响对于术后辅助治疗的选择。最终，关于术后辅助治疗方案的决策需要由多学科团队共同讨论，并综合患者的特点进行个体化制定。建议该患者与主治医生进行进一步的讨论和咨询，共同制定最适合患者的治疗方案。 | The patient has stage IIA rectal cancer with vascular invasion, which is a high-risk factor. Therefore, adjuvant therapy is recommended after surgery. The commonly used regimen is Xelox chemotherapy, also known as CapOx regimen. Specifically, it consists of oxaliplatin 130mg/m2 (body surface area), administered intravenously on day 1, and capecitabine 1250mg/m2 (body surface area), taken orally on days 1-14, once after each meal. There is a drug-free period from day 15 to day 21, followed by the start of the next treatment cycle. Typically, 8 cycles of treatment are administered, with adjustments made to the specific chemotherapy regimen, treatment cycles, and drug dosages based on adverse reactions and tumor recurrence/metastasis. | 该患者为IIA期直肠癌，存在脉管侵犯这一高危因素，因此推荐术后行辅助治疗，一般常规方案为Xelox化疗方案，也称CapOx方案，具体为：奥沙利铂130mg/m^2^（体表面积），第1天静脉输注；卡培他滨1250mg/ m^2^（体表面积），第1-14天口服，早晚餐后各1次。第15-21天停药休息，然后开始下一个治疗周期。一般治疗8个周期，具体化疗方案、治疗周期和用药剂量根据不良反应及肿瘤复发转移等情况进行调整。 |

**Supplementary Table 2**. Example Answers of Report Questions from AI and Non-AI

| RQs | AI answer | | Non-AI answer | |
| --- | --- | --- | --- | --- |
|  | In English | In Chinese | In English | In Chinese |
| RQ5: Prediction of prognosis. | According to the pathological staging (pT3N0M0) in this pathology report, it indicates that the tumor has penetrated the entire wall of the rectum but has not spread to the lymph nodes or distant sites. In this situation, the prognosis for the patient is relatively good. Since the tumor has not spread to distant sites, surgical removal can completely eliminate the tumor, resulting in high cure and survival rates. However, the prognosis is also influenced by various other factors, such as the patient's age, overall health condition, tumor grade, and staging. Therefore, it is best to further conduct clinical evaluation and discussion based on individual circumstances to develop appropriate treatment plans and preventive measures. | 根据这份病理报告中的病理分期（pT3N0M0），表示肿瘤穿透了直肠壁的全层，但没有淋巴结转移，也没有远处转移。在这种情况下，该患者的预后相对较好。因为肿瘤没有远处转移，手术切除可以完全去除肿瘤，有很高的治愈和生存率。不过，预后还受到很多其他因素的影响，如患者年龄、整体健康状况、肿瘤的分级和分期等。因此，最好根据个体情况，进一步进行临床评估和讨论，以制定适合的治疗方案和预防措施。 | According to the literature report, the 5-year survival rate of stage II rectal cancer is approximately 60% to 70%. | 根据文献报道，II期直肠癌的5年生存率约为60%-70%。 |
| RQ6: Advice for follow-up. | According to this pathological report, the following follow-up recommendations are made for postoperative colorectal cancer patients: (1) Within 3 months after the surgery: it is recommended to undergo a comprehensive physical examination, including tumor marker testing (such as CEA); (2) Within 6 months after the surgery: repeat the comprehensive physical examination, including tumor marker testing; (3) In the first year after the surgery: undergo a comprehensive physical examination every 3 months, including tumor marker testing; (4) In the second to third year after the surgery: undergo a comprehensive physical examination every 6 months, including tumor marker testing; (5) From the fourth year after the surgery and onwards: undergo a comprehensive physical examination once a year, including tumor marker testing. In addition to the comprehensive physical examination and tumor marker testing, colonoscopy should also be performed to evaluate the healing of the surgical resection area and exclude the possibility of recurrence or new tumors. Depending on individual circumstances, imaging examinations such as CT scans and MRI may also be required. During the follow-up period, patients should maintain a healthy lifestyle, including balanced diet, regular exercise, smoking cessation, and alcohol limitation. At the same time, pay close attention to any physical discomfort or changes in gastrointestinal symptoms, seek medical attention promptly, and communicate with the doctor. The purpose of follow-up is to detect and manage any potential recurrence or metastasis early, as well as to address complications related to surgery or treatment. It is important to follow the doctor's advice and undergo follow-up on time. | 根据这份病理报告，对于术后结直肠癌患者的随访建议如下：（1）术后3个月内：建议进行全身体检，包括肿瘤标志物检测（如CEA）；（2）术后6个月内：重复进行全身体检，包括肿瘤标志物检测；（3）在术后第1年：每3个月进行一次全身体检，包括肿瘤标志物检测；（4）术后第2-3年：每6个月进行一次全身体检，包括肿瘤标志物检测；（5）术后第4年及以后：每年进行一次全身体检，包括肿瘤标志物检测。除了全身体检和肿瘤标志物检测，还应该进行结直肠镜检查，以评估手术切除区域的愈合情况和排除复发或新发肿瘤的可能性。根据个体情况，可能还需要进行CT扫描、MRI等影像学检查。在随访期间，患者应保持良好的生活方式，包括饮食均衡、定期运动、戒烟限酒等。同时，密切关注身体不适、消化道症状的变化，及时就诊并与医生沟通。随访的目的是早期发现和处理任何可能的复发或转移，以及处理与手术或治疗相关的并发症。请务必遵循医生的建议并按时进行随访。 | According to the guidelines, within 2 years after surgery, patients should undergo regular check-ups every 3-6 months, including colonoscopy, chest, abdomen, and pelvic CT scans, tumor markers (CEA and CA199), and if necessary, PET-CT scans. From 3 to 5 years after surgery, patients should have check-ups every 6-12 months, with the same tests as mentioned above. | 根据指南，术后2年内，患者应每3-6个月进行体检，内容包括:肠镜、胸腹及盆腔CT、肿瘤标志物（CEA和CA199），必要时行PET-CT。术后3-5年患者应每6-12个月进行体检，内容同上。 |

**Supplementary Table 3** Comparison of Readability Scores of Answers from different LLMs

| Sources of answers | | Readability score, mean (SD) | *P* |
| --- | --- | --- | --- |
| All pathology questions | gpt-3.5-turbo | 18.4 (3.5) | 0.184 |
|  | Claude 3 Opus | 16.1 (1.6) |  |
| CQs | gpt-3.5-turbo | 18.1 (2.3) | 0.612 |
|  | Claude 3 Opus | 17.4 (1.1) |  |
| RQs | gpt-3.5-turbo | 18.5 (3.6) | 0.225 |
|  | Claude 3 Opus | 14.9 (1.1) |  |

Note: Readability is measured by the complexity of the text; a higher score signifies a more complex text and thus, lower readability. Statistical analyses between different LLMs were done with the Mann-Whitney *U* test tests.
